# Reduce Calibration Time in Motor Imagery Using Spatially Regularized Symmetric Positives-Definite Matrices Based Classification

**DOI:** 10.3390/s19020379

**Published:** 2019-01-17

**Authors:** Amardeep Singh, Sunil Lal, Hans W. Guesgen

**Affiliations:** School of Fundamental Sciences, Massey University, Manawatu Private Bag 11 222, Palmerston North 4442, New Zealand; S.Lal@massey.ac.nz (S.L.); H.W.Guesgen@massey.ac.nz (H.W.G.)

**Keywords:** motor imagery, symmetric positives definite matrices, brain-computer interface (BCI), electroencephalography (EEG), Riemannian manifold

## Abstract

Electroencephalogram (EEG) based motor imagery brain–computer interface (BCI) requires large number of subject specific training trials to calibrate the system for a new subject. This results in long calibration time that limits the BCI usage in practice. One major challenge in the development of a brain–computer interface is to reduce calibration time or completely eliminate it. To address this problem, existing approaches use covariance matrices of electroencephalography (EEG) trials as descriptors for decoding BCI but do not consider the geometry of the covariance matrices, which lies in the space of Symmetric Positive Definite (SPD) matrices. This inevitably limits their performance. We focus on reducing calibration time by introducing SPD based classification approach. However, SPD-based classification has limited applicability in small training sets because the dimensionality of covariance matrices is large in proportion to the number of trials. To overcome this drawback, our paper proposes a new framework that transforms SPD matrices in lower dimension through spatial filter regularized by prior information of EEG channels. The efficacy of the proposed approach was validated on the small sample scenario through Dataset IVa from BCI Competition III. The proposed approach achieved mean accuracy of 86.13% and mean kappa of 0.72 on Dataset IVa. The proposed method outperformed other approaches in existing studies on Dataset IVa. Finally, to ensure the robustness of the proposed method, we evaluated it on Dataset IIIa from BCI Competition III and Dataset IIa from BCI Competition IV. The proposed method achieved mean accuracy 92.22% and 81.21% on Dataset IIIa and Dataset IIa, respectively.

## 1. Introduction

Electroencephalogram (EEG) based brain–computer interfaces (BCI) detect neural activity from brain scalp and translate them into control commands for external devices [1]. EEG based BCI systems can be categorized as exogenous or endogenous, according to paradigm used to generate neural activity [2]. An exogenous BCI derives its output from neural activity (EEG signals) generated due to attentional selection of an external stimulus among many [3]. An endogenous BCI derives its outputs from oscillatory neural activity, which is spontaneously controlled by the user [3]. Endogenous BCI does not require external stimulus to generate specific neural pattern for BCI, in fact the user can control BCI system voluntarily. Motor imagery (MI) is one such endogenous BCI paradigm where neural activity is generated at the sensorimotor cortex due to the kinaesthetic imagination of a body part (left/right hand) movement [4].

During MI, there is a rhythmic power decrease or increase in measured EEG signals from the sensorimotor cortex. These phenomena are also known as event related desynchronization (ERD) and event related synchronization (ERS), respectively [5]. BCIs distinguish different MI tasks through spatial and temporal properties of measured EEG signals [6]. Therefore, to increase the spatial and temporal resolution, electroencephalogram (EEG) signals are recorded with multi-channel electrodes system with high sampling rate. This results in high-dimensional signals.

MI-based BCIs are indeed very promising for people suffering from neuromuscular disorder, but still lack adoption as access modalities outside laboratories. The main reason that prevents MI-based BCIs from widely being used is high performance variations among and within subjects. These performance variations are due to change in the external (user’s muscle movements, recording condition and machine related causes) and internal (user’s cognitive state of mind) state of the user [7]. Therefore, it requires extensive training compared to exogenous BCI systems. During training and calibration phase, new subjects learn to voluntarily regulate oscillatory EEG pattern and training trials are collected to obtain discriminative features that are fed into machine learning algorithms for MI classification [8].

The standard feature extraction techniques for motor imagery use covariance matrices of trials. One such technique is common spatial pattern (CSP) that aims to determine optimal spatial filters that discriminate two MI task (left/right) [9]. CSP requires large number of subject specific calibration trial sessions to achieve good MI classification. These calibration sessions are very time consuming and not user-friendly. Thus, it is desirable to reduce or remove the calibration entirely.

However, in the case of a small EEG calibration trials set, these covariance matrices poorly estimate MI and therefore lead to poor performance of CSP. To address this, Lu et al. [10] proposed Regularized CSP, which uses other subjects’ trials to construct MI classes spatial covariance matrices for new target subjects that will be used to extract CSP features. In the same vein, Dai et al. [11] also employed transfer learning technique to learn domain invariant CSP features from source and target subjects. Both approaches rely on other subjects (source) to subject (target) transfer learning, which, in the worst case, might hurt the performance of the target subject. This situation is often called negative transfer [12].

Unlike the above methods, Arvaneh et al. [13] suggested a technique that does not rely on source subjects trials. Rather, this approach optimizes obtained CSP filters by using channels from brain regions that have high variances between MI classes, and attenuates the noisy channels from regions with low and irregular variances. Similarly, Lotte et al. [14] used spatial information of electrodes as prior knowledge to regularize objective function of the CSP algorithm to obtain spatial filters. In similar manner, Park and Chung [15] used electrodes from certain brain regions to extracted diverse CSP features and obtain high accuracy compared to standard CSP under small training samples (trials).

The efficiency of spatial filter is sensitive to individual’s temporal and frequency characteristics. To address subject specific frequency characteristics issue, Ang et al. [9] proposed filter bank CSP (FBCSP) that uses multiple bandpass filters to extract CSP features. FBCSP may lose important frequency information, as it uses fixed partition of the frequency (frequency width of 4 Hz, varying from 4 Hz to 30 Hz). To address this problem, Yang et al. [16] proposed CSP feature extraction based on varying partition of the frequency bands with different bandwidth to cover as many bands as possible. In a similar way, Park and Lee [17] extended FBCSP by regularizing CSP features obtained from multiple filter banks. They used other subjects’ trials covariance matrices to regularize filter bank CSP features. Zhang et al. [18] proposed a method that simultaneously optimizes filter bands and time window used to obtain CSP features to further boost classification accuracy of MI. Filter bank-based methods result in a high dimensional CSP feature set, therefore requires a feature selection algorithm to select discriminative CSP features for MI classification. To address the feature selection problem, Selim et al. [19] used bio-inspired optimization algorithm for feature selection. They also selected optimal time interval for each subject to extract CSP features. Unlike the above methods, Tang et al. [20] used a convolutional neural network model to classify MI tasks based on spatiotemporal characteristics of EEG. Furthermore, Tabar and Halici [21] combined convolutional neural network and stacked autoencoders to classify EEG Motor Imagery signals.

All methods discussed above use covariance matrices of trials to extract CSP features (log variance) into a vector in Euclidean space. Furthermore, pattern recognition metrics used to classify features also lies in Euclidean space. As covariance matrices lie in the symmetrical positive definite (SPD) matrices manifold, these methods fail to notice distinct characteristic of EEG data such as their interrelation across the manifold dimensions [22].

The effectiveness of data treatment based on the concept of geometrical properties was proved by Barachant et al. [23]. They proposed minimum distance to Riemannian mean (MDRM) classification technique that adopts Riemannian distance as pattern recognition metric to classify test trials. MDRM outperforms standard CSP approach, but performance of MDRM declines as the size of covariance matrices grows. Under small training set, the size of covariance matrices are larger than the number of trials. Therefore, MDRM algorithms encounter the curse of dimensionality problem [24].

To address dimensionality problem, Horev et al. [25] adapted PCA to the space of SPD matrices, which conserves more data variance and maps covariance matrices to a lower-dimensional SPD manifold. In a similar manner, Harandi et al. [26] learned mapping that maximizes the geodesic distances between inter-class samples and simultaneously minimizes the distances between intra-class samples. This was done via optimization on Grassmann manifolds. This algorithm tries to preserve the local structure of the data by preserving distance to local means, considers the geometry of SPD matrices, provides an implicit mapping and applies the supervised information for embedding to lower-dimensional space. Furthermore, Davoudi et al. [24] extended Harandi’s work by proposing another dimensionality reduction algorithm for the manifold of SPD matrices which preserves the local structure of data by preserving distance to local mean (DPLM). This algorithm can work in a supervised (sDPLM) or unsupervised (uDPLM) manner and projects a high-dimensional SPD manifold to a lower-dimensional one. In the same vein, Kumar et al. [27] also addressed dimensionality issue of covariance matrices by using spatial filtering. The drawback of this method is that it requires many subject-specific trials to optimize spatial filter performance. In this paper, we propose a method that uses the best of both Euclidean and SPD space. We use prior information of EEG electrodes to obtain spatial filter that transform sample covariance matrices (SCM) into lower dimension. Then, Riemannian distance is used as pattern recognition metric for classification as it is invariant to any linear invertible transformation [28].

The rest of the paper is organized as follows. In Section 2, we review the space of SPD matrices and MDRM classification approach. Section 3 presents our proposed SR-MDRM classification approach. Section 4 describes the experiment as well as datasets. In Section 5, we discuss and compare results of the experiment with existing studies. Section 6 draws the conclusions regarding proposed approach.

## 2. Geometry of SPD Matrices

An *n* × *n* square matrix *C* falls in the space of symmetric positive definite SPD(n) if $C=C^{T}$, $u^{T}Cu>0$ and ∀u≠0. Equivalently, SPD matrices have the following properties:

1. $\forall C\in SPD\left(n\right),C^{-1}\in SPD\left(n\right)$ i.e., SPD matrices are invertible.
2. ∀C∈SPD(n), eigenvalues are positive i.e., λ(C)>0.

$C^{k}$, log(C) and exp(C) operation on $C\in \{SPD\left(n\right),R^{n\times n}\}$ are defined by its eigenvalues ($\lambda_{i}$) and eigenvector *U* as:

(1) $$C^{k}=Udiag\left(\left[\lambda_{1}^{k},\ldots ,\lambda_{n}^{k}\right]\right)U^{T}$$

(2) $$\log\left(C\right)=Udiag\left(\left[log\left(\lambda_{1}\right),\ldots ,log\left(\lambda_{n}\right)\right]\right)U^{T}$$

(3) $$\exp\left(C\right)=Udiag\left(\left[exp\left(\lambda_{1}\right),\ldots ,exp\left(\lambda_{n}\right)\right]\right)U^{T}$$

Covariance matrices of EEG trial lies in symmetric positive definite matrices manifold [29]. Covariance matrices hold spatial information for EEG trial and can directly be used for classification. SPD matrices lie on a differentiable Riemannian manifold. Therefore, all properties of the Riemannian manifold are applicable to SPD matrices.

### 2.1. Riemannian Natural Manifold

**Figure 1:**
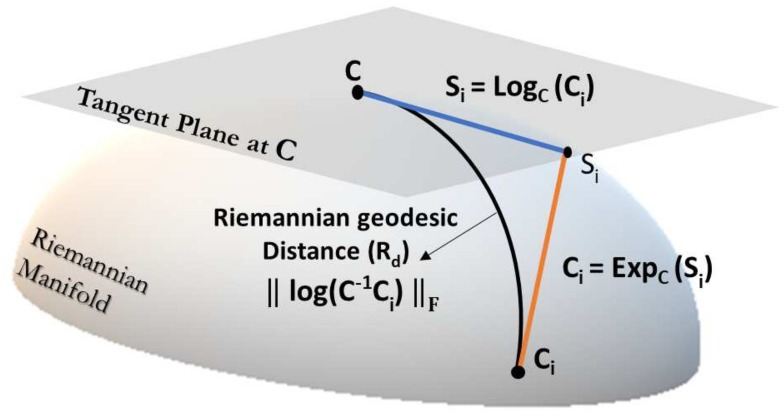
Illustration of Tangent space at point *C*. Tangent vector $S_{i}$ is the projection of $C_{i}$ and $R_{d}$ is the geodesic between *C* and $C_{i}$ [4].

A Riemannian manifold is the topological space [22] where each point *C*’s derivative lies in a Euclidean vector space T(N) that is tangent at that point, as shown in Figure 1.

In the case of Riemannian manifold R(N), the tangent space T(N) is a space of symmetric matrices ($S\left(n\right),S^{T}=S$).

A point (matrix) $C_{i}$ is projected to tangent space T(N) using logarithmic mapping $Log_{C}\left(C_{i}\right)$ as

(4) $$S_{i}\in T(N)=Log_{C}\left(C_{i}\right)=C^{1/2}logm\left(C^{-1/2}C_{i}C^{-1/2}\right)C^{1/2}$$

where *C* is a reference point in the manifold R(N) where the tangent plane is mapped and logm(.) is logarithm of SPD matrix given in Equation (2). Furthermore, the tangent vector $S_{i}$ from tangent space T(N) is projected back to manifold R(N) using exponential mapping $Exp_{C}\left(S_{i}\right)$ given by

(5) $$C_{i}\in R(N)=Exp_{C}\left(S_{i}\right)=C^{1/2}expm\left(C^{-1/2}S_{i}C^{1/2}\right)C^{1/2}$$

where expm(.) is exponential of SPD matrix, as shown in Equation (3).

### 2.2. Riemannian Distance

Riemannian distance is a unique and shortest (geodesic) curve connecting two points C1 and C2 in the Riemannian manifold R(N). It is given by

(6) $$R_{d}\left(C_{1},C_{2}\right)={∥\log\left(C_{1}^{-1/2}C_{2}C_{1}^{-1/2}\right)∥}_{F}={\left(\sum_{i=1}^{n}\log^{2}\lambda_{i}\right)}^{1/2}$$

where ${∥.∥}_{F}$ is the Frobenius norm and $\lambda_{i}$’s are the positive eigenvalues of $C_{1}^{-1/2}C_{2}C_{1}^{-1/2}$. The Riemannian distance $R_{d}\left(C_{1},C_{2}\right)$ is invariant to any linear invertible transformation [28]:

(7) $$R_{d}\left(A^{T}C_{1}A,A^{T}C_{2}A\right)=R_{d}\left(C_{1},C_{2}\right)$$

where *A* is an invertible matrix. The Riemannian distance between two points in manifold R(N) can be approximated in tangent space T(N) by approximating the distance between projected tangent vectors through a reference point *C*. To obtain a good approximation of the Riemannian (geodesic) distance, reference point *C* needs to be close to two points in the manifold R(N). Usually, the Riemannian mean $\Pi_{R}$ is the most suitable choice for the reference point.

### 2.3. Riemannian Mean (Choice of Reference Point)

The Riemannian mean is a unique point in the manifold R(N) that gives better local approximation of the manifold when it is mapped to the tangent space [4]. It minimizes the sum of squared Riemannian distances. It is also referred to as the geometric mean of SPD matrices and is given by

(8) $$\Pi_{R}\left(C_{1},\ldots ,C_{N}\right)=\underset{C\in SPD(N)}{\arg\min}\sum_{i=1}^{N}{R_{d}}^{2}\left(C_{i},C\right)$$

The mean of *N* SPD matrices such as EEG trials covariance matrices keeps shifting due to the non-stationarity of EEG signals. Therefore, it needs to be iteratively computed whenever any new trials are collected. The computation of the Riemannian mean $\Pi_{R}$ goes through the following steps until it converges. Firstly, covariance matrices are projected in the tangent space using a Riemannian log map. Secondly, the tangent space of Riemannian manifold is Euclidean, therefore arithmetic mean $\Pi_{T}$ can be easily computed. The arithmetic mean $\Pi_{T}$ is a point *C* that minimizes the sum of squared Euclidian distances $T_{d}$ between projected SPD matrices and is given by

(9) $$\Pi_{T}\left(C_{1},\ldots ,C_{N}\right)=\underset{C\in SPD(N)}{\arg\min}\sum_{i=1}^{N}{T_{d}}^{2}\left(C_{i},C\right)$$

Finally, the arithmetic mean is projected back to the manifold using exponential mapping.

### 2.4. Minimum Distance to Riemannian Mean (MDRM)

MDRM is a classification approach that uses the Riemannian mean of each class and its Riemannian distance to test covariance matrix of the trial to predict a label for it. In this approach, the Riemannian mean is calculated for each class using its labeled training trials, and then the Riemannian distance of each class is calculated with respect to test trial’s covariance matrix. The class mean that is closest to test trial covariance becomes the trial’s label.

(10) $$pred\left(C_{x}\right)=\underset{\varphi =1,2..C}{\arg\min}R_{d}\left(C_{x},C_{\mu }\right)$$

where $C_{x}$ is the covariance matrix of the test trial, $C_{\mu }$ is the Riemannian mean of Class φ and $pred(C_{x})$ is the prediction of its class label. The MDRM approach is not robust to noise [24], therefore, some filtering over SPD matrices is required. Barachant et al. [30] suggested geodesic filtered MDM (FGMDM) approach, which computes set of filters by applying a supervised Fisher geodesic discriminant analysis (FGDA) to the tangent (Euclidean) space projection of covariance matrices. The obtained filters are applied through geodesic filtering approach [30] over SPD matrices. This filtering operation do not change any dimension of the SPD matrices. Finally, the filtered SPD matrices are used for MDRM classification.

## 3. Methodology

**Figure 2:**
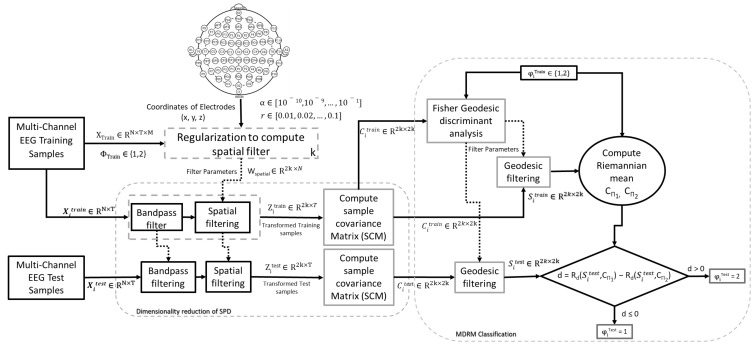
Framework for proposed approach.

The conceptual framework of our proposed methodology is shown in Figure 2. EEG signals are often divided into trials based on the label given during training phase. Let $X_{i}$∈ ($R^{N\times T}$) be a bandpassed EEG trial where *N* is number of electrodes and *T* is sampled time points in the trials and trial labels $\varphi_{i}\in \left(1,2\right)$. Therefore, the training set can be given as ${\left\{X_{i},\varphi_{i}\right\}}_{i=1}^{M}$ where *M* is the total number of training trials. The covariance matrix $C_{i}$ for trial $X_{i}$ is calculated as follows:

(11) $$C_{i}=\frac{X_{i}X_{i}^{T}}{tr(X_{i}X_{i}^{T})}$$

where tr(.) denotes the trace operator of the matrix, and the superscript *T* denotes the transpose of the matrix. The sample covariance matrix for class can be obtained by taking sum of sample covariance matrices for *M* trials that belong to it. It is calculated as follows:

(12) $$C_{\varphi }=\sum_{m=1}^{M}C_{\left(\varphi ,m\right)}$$

where *M* is the total trial number of each class, and *m* is the index of the trial (m≤M). φ denotes the class index, and we consider only two classes (φ∈{1,2}) in this paper. Spatial filters *w* through CSP are obtained by extremizing the following function:

(13) $$J\left(w\right)=\frac{w^{T}C_{1}w}{w^{T}C_{2}w}$$

This is an optimization problem that can be solved by Lagrange multiplier method using Equation (14):

(14) $$L\left(\lambda ,w\right)=w^{T}C_{1}w-\lambda \left(w^{T}C_{2}w-1\right)$$

The filters *w* extremizing *L* are such that the derivative of *L* with respect to *w* equals 0:

(15) $$\begin{array}{c} \frac{\partial L}{\partial w}=2w^{T}C_{1}-2\lambda w^{T}C_{2}=0\\ \Leftrightarrow C_{1}w=\lambda C_{2}w\\ \Leftrightarrow C_{2}^{-1}C_{1}w=\lambda w \end{array}$$

Equation (15) is a standard eigenvalue problem. To get optimal results, first and last *k* eigenvectors of $C_{2}^{-1}C_{1}$ are used as the spatial filters *w*. From a neuro-physiological point of view, neighboring brain cells tend to function similarly, so neighboring electrodes should measure similar brain activity signals [31]. Thus, we can expect that neighboring channels of the spatial filter should have similar weights (i.e., smooth spatial filter). To obtain smooth spatial filter, we use the spatial information of electrodes as a prior knowledge [14] to penalize objective function of CSP algorithm. Smooth spatial filters *w* can be obtained by extremizing the following functions:

(16) $$\begin{array}{c} J_{P_{1}}\left(w\right)=\frac{w^{T}C_{1}w}{w^{T}C_{2}w+\alpha P\left(w\right)}\;\mathrm{and}\;J_{P_{2}}\left(w\right)=\frac{w^{T}C_{2}w}{w^{T}C_{1}w+\alpha P\left(w\right)} \end{array}$$

The penalty term P(w) measures the spatial smoothness of the spatial filters *w*, where $P\left(w\right)=w^{T}Kw$ with K=D−G. *G* is a Gaussian Kernel such that $G_{ij}=\exp-\frac{1}{2}\left(\frac{{\left|\right|v_{i}-v_{j}\left|\right|}^{2}}{r^{2}}\right)$, with $v_{i}$ a vector containing 3D coordinates of the *i*th electrode. *D* is a diagonal matrix such as $D_{ii}=\sum_{j}G_{ij}$. Therefore, $w^{T}Kw=w^{T}\left(D-G\right)w=\sum_{i,j}G_{ij}{\left(w_{i}-w_{j}\right)}^{2}$. There are two hyperparameters (r,α) in regularized objective function. The first hyperparameter *r* defines how far two electrodes can be to be still considered as close to each other and the second hyperparameter α defines the level of spatial smoothness the filters should reach. Equation (16) becomes:

(17) $$\begin{array}{c} J_{P_{1}}\left(w\right)=\frac{w^{T}C_{1}w}{w^{T}C_{2}w+\alpha w^{T}Kw}\;\mathrm{and}\;J_{P_{2}}\left(w\right)=\frac{w^{T}C_{2}w}{w^{T}C_{1}w+\alpha w^{T}Kw} \end{array}$$

using Lagrangian multiplier method, the solution is obtained as,

(18) $$\begin{array}{c} M_{1}={\left(C_{2}+\alpha K\right)}^{-1}C_{1}\;\mathrm{and}\;M_{2}={\left(C_{1}+\alpha K\right)}^{-1}C_{2} \end{array}$$

We construct the projection matrix $W_{p}\in R^{2N\times N}$ using Equation (19)

(19) $$W_{p}=\left[M_{1}M_{2}\right]$$

The EEG signal trial is transformed with $W_{spatial}$ made from the first and last *k* columns of $W_{p}$ by using Equation (20)

(20) $$Z_{i}=W_{spatial}X_{i}$$

where $Z_{i}\in R^{2k\times T}$ is transformed signal corresponding to $X_{i}$. Sample covariance matrices $C_{i}^{train}$ of filtered EEG trials $Z_{i}$ from the target’s training set are calculated using Equation (11). These SCMs $C_{i}^{train}$ are used to obtain FGDA filter for geodesic filtering by using an algorithm, as mentioned in [30]. After geodesic filtering, filtered SCMs ($S_{i}^{Train}$ and $S_{i}^{Test}$) for target subject’s training and test trials are obtained. Finally, filtered SCMs of the target subject’s training set ($S_{i}^{Train}$) are used in calculating Riemannian mean $C_{\Pi_{R}\varphi }$ for both motor imagery classes. These Riemannian means are used for MDRM classification [30] of test trials $S_{i}^{Test}$.

## 4. Data and Experiment

To assess the performance of our method for small training setting, we used the EEG Dataset IVa from BCI Competition III. Furthermore, we compared it with existing methods designed for small training set scenario. To confirm the robustness of proposed approach, we evaluated it over two publicly available datasets with a different number of EEG channels from BCI competition. A summary of the three datasets is given in Table 1.

### 4.1. Dataset IVa, BCI Competition III

Dataset IVa [32] contains EEG signals of binary (right hand and foot) motor imagery tasks from five healthy subjects. EEG signals were recorded using 118 electrodes at 100 Hz sampling rate. There are a total 280 trials per subject that are unevenly divided into training and testing set for each subjects, as shown in Table 1.

### 4.2. Dataset IIIa, BCI Competition III

Dataset IIIa [32] comprises of EEG signals of multi-class (right hand, left hand, tongue and foot) motor imagery (MI) tasks from three subjects (“k3b”, “k6b” and “l1b”). EEG signals were sampled at 250 Hz rate and recorded using 60 electrodes. In this study, we used EEG signals from trials corresponding to binary MI class (left/right). There are total 180 trials for subject “k3b” and 120 trials for subjects “k6b” and “lib”, respectively.

### 4.3. Dataset IIa, BCI Competition IV

Dataset IIa [33] contains of data recorded from 22 EEG channels and 3 EOG channels at sampling rate of 250 Hz. Dataset IIa contains multi class EEG signals from nine subjects, namely A01–A09. In this experiment, we considered data collected from 22 EEG electrodes corresponding to left and right MI class from each of nine subjects. Table 1 shows number of training and testing trials for all subjects.

### 4.4. Experimental Setup

This study was carried out using a Windows 10 computer with specification Intel (R) Core *™* i5–6500 CPU @3.20 GHz with 8 GB RAM. All conventional methods (CSP and MDRM) and proposed algorithm were designed and tested in Matlab R2018a.

The study comprised six steps. Firstly, we used a time segment from 0.5 s to 2.5 s after the visual cue for all the datasets considered for this study [34,35]. Thus, trials respective to Dataset IVa, Dataset IIIa and Dataset IIa comprised 200, 500 and 500 sampled time points. Secondly, all trials were filtered in frequency range within 7–30 Hz through fifth order Butterworth bandpass filter. This frequency band was selected as it comprises the alpha and beta frequency bands, which have been shown to be most important for MI task classification [36,37]. Thirdly, spatial filters were learned using regularization parameters $\alpha \in [10^{-10},10^{-9},\ldots ,10^{-1}]$ and r∈[0.01,0.02,Ȧ,0.09,0.1]. In the fourth step, the EEG signals were transformed into lower dimension using regularized spatial filter. In the fifth step, covariance matrices for training trials were employed to obtain FGDA filters. Lastly, after geodesic filtering, Riemannian mean for each MI class was calculated using training trials covariance matrices and labels were assigned to test trials based on their distance from the Riemannian mean of MI classes. For CSP and MDRM, we used the same time-segment, filter order and frequency band as described for the proposed method.

### 4.5. Evaluation Metrics

To evaluate the performance of proposed method, we used classification accuracy and kappa coefficient as evaluation metrics. In binary classification case, accuracy can be calculated as described in Equation (21).

(21) $$Accuracy=\frac{a+b}{a+b+c+d}$$

where *a* is the number of positive samples correctly identified, *b* is the number of negative samples correctly identified, *c* is the number of negative cases incorrectly identified, and *d* is the number of positive cases incorrectly identified. Kappa coefficient compares the accuracy of the system to the accuracy of a random system. It is defined as

(22) $$kappa=\frac{observeredAccuracy-randomAccuracy}{1-randomAccuracy}$$

where random accuracy is given by

(23) $$randomAccuracy=\frac{\left(b+c)*(b+d)+(d+a)*(c+a\right)}{{\left(a+b+c+d\right)}^{2}}$$

## 5. Results and Discussion

We evaluated the performance of the proposed approach (SR-MDRM) on the three datasets, and compared it with conventional (CSP and MDRM) methods as well as benchmark results reported in the literature. Table 2 shows regularization parameters used in SR-MDRM classification for all subjects belonging to different datasets.

### 5.1. Dataset IVa, BCI Competition III

Table 3 shows the classification accuracy proposed method, winner of BCI Competition III on Dataset IVa, CSP method and other benchmark results reported in the literature on Dataset IVa.

As shown in Table 3, our method outperformed the existing studies in the literature, except for the winner. In this study, we used same approach for all subjects; on the contrary, winner [32] did not use the same approach for all subjects. Wang et al. (winner) [32] used an ensemble classifier based on CSP, autoregressive (AR) and Temporal waves of readiness potential (RP). Only CSP method was applied for subject al, aw and ay but for subject aa and av combination of all three methods (CSP–AR–RP) was used. Moreover, for subjects with fewer training data (aw and ay), they used former classified test sample as extended training samples, whereas our proposed approach used only training samples even for subjects with limited training trials. Therefore, it is unfair to compare our simple methods with the first winner.

Selim et al. [19] used subject specific optimal time interval for CSP feature extraction. Furthermore, they used hybrid bio-inspired algorithms for feature selection and classifier optimization. They achieved 85% classification accuracy, which is slightly ( 1.13% ) less than the proposed approach. One drawback of this approach is that the classifier optimization takes a very long time. Park and Chung [15] used a set of various local channels region to extract CSP features. They used eigenvalue disparity score to select CSP features from the local channel region and support vector machine (SVM) classifier to classify extracted features. They obtained 84.86% accuracy, which is less than the proposed approach by 1.67%. Park and Lee [17] (SBRCSP) focused on regularizing CSP features from filter bank using other subjects training trials. Their results were less than the proposed approach by 3.44%. In the same vein, Dai et al. [11] implemented a “Transfer Kernel CSP” (TKCSP) approach to learn a domain-invariant kernel by directly matching distributions of source subjects and target subjects. Similar to our approach, they employed all 118 channels to obtain 82.69% which is less than our approach by 6.96. Both TKCSP and SBRCSP have the same drawback, as they rely on other subjects’ (source) training trials.

Selim et al. [39] used root mean square (RMS) features for LDA classifier to obtain 78.77% accuracy with 7.36% less than that of proposed approach. Lotte and Guan [14] penalized CSP objective function to obtain smooth filters to extract features and achieved 78.63%, which is less than proposed approach by 7.50%. Similarly, Arvaneh et al. [13] implemented “Spatially Sparsed CSP” (SSCSP) filters to extract CSP features. Their results were less than the proposed approach by 12.63%. Belwafi et al. [38] used weighted overlap-add (WOLA) algorithm to perform dynamic filtering of EEG-signals for filter bank CSP method. Their method achieved 67.29% classification accuracy, which is less than our approach by 18.85%. Our method improved the mean classification accuracy by 19.85% compared to CSP method.

Our proposed method shows the highest classification accuracy for subject al. Lotte and Guan [14] identified subject av as BCI illiterate with CSP method because their performance was below 55% (close to random). However, with SR-MDRM, subject av achieved 73.46% classification accuracy. Subject av would no longer be identified as illiterate. Subject ay’s accuracy improved drastically with only 28 training trials. Thus, we might hypothesize that adding spatial prior along with geometry based classification increases accuracy despite the limited amount of training data.

**Figure 3:**
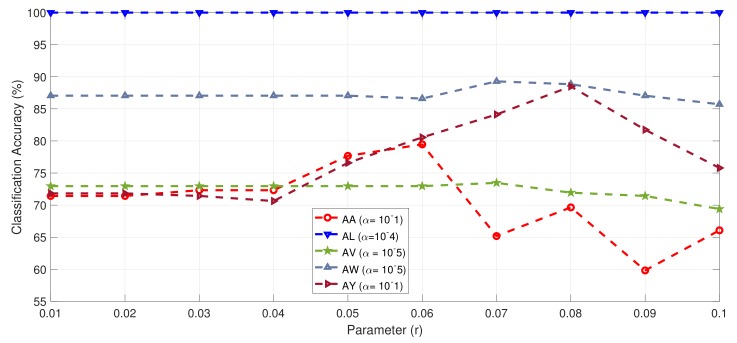
Classification accuracy of all subjects from Dataset IVa with respect to parameter *r* and best value of α.

The regularization parameter *r* controls the trade off between accuracy and filters sparsity. Therefore, the optimal *r* value must be selected to increase the accuracy. Figure 3 shows the effect of *r* values on the classification accuracy values of all subjects with fixed (best) α value. Subject al reached maximum accuracy independent from value of *r* parameter. This is because al had sufficient training data. Other subjects performance showed dependence on the value of *r* and reached maximum accuracy for particular *r* and α value.

Table 4 shows performance of SPD manifold based classification methods for all the subjects in kappa values. As shown in Table 4, our method outperformed all existing methods. In addition, SR-MDRM obtained highest kappa value for subjects al, av and aw. As Dataset IVa represents a small sample setting, results obtained on it signify that SR-MDRM is suitable for small sample scenarios.

### 5.2. Dataset IIIa, BCI Competition III

Dataset IIIa is also a good test environment for proposed approach, as it also has limited training samples and high EEG signals dimensionality. Table 5 presents classification accuracy of proposed method and other existing methods on Dataset IIIa. As shown in Table 5, SR-MDRM method on average improved performance by 4.59%, 7.97%, 8.70%, 9.26%, 10.56%, and 11.11% compared to Tgcsp [18], Wola-Csp [38], Csp, Horev-Mdrm [25], Mdrm [23] and Srcsp [35] methods, respectively.

**Figure 4:**
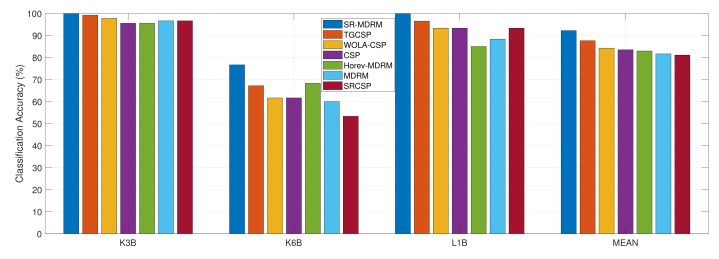
Classification accuracy of the proposed approach and other MI classification approaches on Dataset IIIa, BCI Competition III.

In Figure 4, the SR-MDRM method shows a higher mean classification accuracy than the six other methods. In addition, the SR-MDRM method shows the highest classification accuracy for individual subjects. That is, Figure 4 clearly shows that the SR-MDRM method is more efficient for binary motor imagery classification than the other six methods.

Zhang et al. [18] proposed temporally constrained sparse group spatial pattern (TSGSP) method; their performance was slightly less than our method. In their study, they simultaneous optimized filter bands and time window to extract CSP features for classification to obtain mean accuracy of 87.63%. Dataset IIIa is recorded with (60) electrodes, thus covariance matrices dimensionality is less compared to Dataset IVa (118×118). MDRM method’s performance improved due to small size of covariance matrices. As shown in Figure 4, it is marginally less (1.3%) than Horev’s MDRM [25] method, which adapted PCA to map covariance matrices to a lower-dimensional SPD manifold. Interestingly, standard CSP performed better than spatially regularized CSP method proposed [35] on Dataset IIIa.

**Figure 5:**
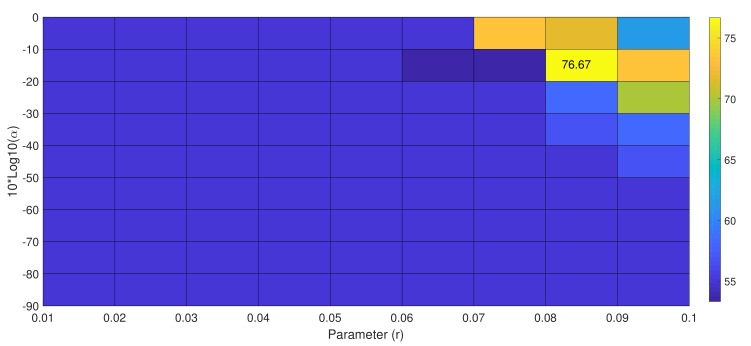
Classification accuracy according to parameter *r* and $10\log_{10}\left(\alpha \right)$ of the proposed approach on subject K6B from Dataset IIIa, BCI Competition III.

Subject k6b’s performance improved drastically with our proposed approach. Subject k6b gained 9.47% classification accuracy more then the state-of-the-art method tgcsp[18]. Figure 5 shows the classification accuracy values of subject k6b according to the parameter *r* and α, respectively. Subject k6b reached maximum accuracy at $\alpha =(10^{-3})$ and r=0.08 values. It proves our hypothesis that spatial prior and geometry based treatment of data helps achieve the highest classification accuracy under small training sample.

### 5.3. Dataset IIa, BCI Competition IV

Dataset IIa has sufficient training trails per subjects and EEG signals are low dimensional (22 channels), as shown in Table 1. It is a good test environment to check for the robustness of our proposed approach under low dimensional and sufficient training samples.

Table 6 shows the classification accuracy of existing methods and proposed method (SR-MDRM) on Dataset IVa. For subjects A02, A03, A04 and A07, the proposed method achieved highest accuracy compared to existing methods in the literature. Similar to our proposed approach, Gaur et al. [40] used Riemannian geometry to classify features obtained through subject specific multivariate empirical mode decomposition method (SS-MEMD). They achieved higher accuracy for subject A09 and mean accuracy was slightly less than proposed approach. Due to lower dimensionality of covariance matrices in Dataset IIa, MDRM method outperformed other methods for subjects A01 and A05.

As shown in Table 6 the SR-MDRM method improves the mean classification accuracy by 1.29%, 1.77%, 2.37%, 2.44%, 3.21%, 7.38% and 6.30% in comparison with SS-MEMDBF, MDRM, WOLA-CSP, SRCSP, CSP, ${\mathrm{TLCSP}}_{1}$, and ${\mathrm{TLCSP}}_{2}$, respectively.

## 6. Conclusions

We propose spatially regularized Symmetric positive definite (SPD) matrices based motor imagery classification method. This method incorporates prior information of EEG electrodes to obtain spatial filters that transform sample covariance matrices into lower dimension and maximize the variance between two motor imagery task in small sample setting. The proposed method takes advantage of geometrical properties of covariance matrices by employing Riemannian distance as pattern recognition metric for classification as it is invariant to any linear invertible transformation. The efficacy of the proposed approach was validated on three public datasets from BCI competition. Our proposed method transcends other approaches in existing studies on all three datasets. 

## Figures and Tables

**Table 1:** Summary of Dataset IVa, Dataset IIIa and Dataset IIa from BCI competitions (BCIC).

BCI Competition (BCI-C)	BCI-C III								BCI-C IV		
Dataset	Dataset IVa					Dataset IIIa			Dataset IIa		
Electrodes	118					60			22		
Sampling Rate	100 Hz					250 Hz			250 Hz		
Subject	aa	al	av	aw	ay	k3b	k6b	l1b	A01–A09		
Train	168	224	84	56	28	90	60	60	144		
Test	112	56	196	224	252	90	60	60	144		

**Table 2:** Regularization Parameters α,*r* of SR-MDRM for all subjects belonging to different datasets.

Parameters	Dataset IVa					Dataset IIIa			Dataset IIa								
	aa	al	av	aw	ay	k3b	k6b	l1b	A01	A02	A03	A04	A05	A06	A07	A08	A09
α	$10^{-1}$	$10^{-4}$	$10^{-5}$	$10^{-5}$	$10^{-1}$	$10^{-4}$	$10^{-3}$	$10^{-10}$	$10^{-4}$	$10^{-2}$	$10^{-3}$	$10^{-3}$	$10^{-4}$	$10^{-2}$	$10^{-3}$	$10^{-10}$	$10^{-10}$
r	0.06	all r values	0.07	0.07	0.08	0.1	0.08	0.01	0.06	0.07	0.05	0.09	0.07	0.04	0.06	0.01	0.01

**Table 3:** Classification accuracy (Mean and Standard deviation in percent) of the proposed approach and other MI classification approaches on Dataset IVa, BCI Competition III.

Studies	Methods	Year	aa	al	av	aw	ay	Mean	Std
Conventional Method	Csp		66.07	96.43	47.45	71.88	49.6	66.28	19.83
Belwafi et al. [38]	Wola-Csp	2018	66.07	96.07	52.14	71.43	50	67.29	18.54
Arvaneh et al. [13]	Sscsp	2011	72.32	96.42	54.10	70.53	73.41	73.35	15.09
Lotte and Guan [14]	Srcsp	2010	72.32	96.43	60.20	77.68	86.51	78.63	13.77
Selim et al. [39]	Rms/Lda	2016	69.64	89.29	59.18	88.84	86.90	78.77	13.65
Dai et al. [11]	Tkcsp	2018	68.10	93.88	68.47	88.40	74.93	79.17	11.78
Park and Lee [17]	Sbrcsp	2017	86.61	98.21	63.78	89.05	73.81	82.69	13.53
Park and Chung [15]	Sss-Csp	2018	74.11	100	67.78	90.07	89.29	84.46	13.05
Selim et al. [19]	csp/am-ba-svm	2018	86.61	100	66.84	90.63	80.95	85.00	12.30
Proposed Method	sr-mdrm		79.46	100	73.46	89.28	88.49	86.13	10.15
Wang et al. [32]	Winner		96.00	100	81.00	100	98.00	94.20	8

**Table 4:** The performance of proposed approach and existing Riemannian geometry based approaches on Dataset IVa of BCI Competition III in terms of kappa values.

Studies	Year	aa	al	av	aw	ay	Mean
Barachant et al. [23] Mdrm		0.22	0.86	0.25	0.13	0	0.29
Harandi et al. [26] Mdrm	2014	0.23	1.00	0.40	0.53	0.82	0.59
Horev et al. [25] Mdrm	2017	0.62	0.96	0.42	0.68	0.60	0.65
Davoudi et al. [24]-uDplm	2017	0.57	1.00	0.39	0.64	0.72	0.66
Davoudi et al. [24]-sDplm	2017	0.63	1.00	0.46	0.66	0.78	0.70
sr-Mdrm		0.58	1	0.47	0.79	0.77	0.72

**Table 5:** Classification accuracy (Mean and Standard deviation in percent)of the proposed approach and other MI classification approaches on Dataset IIIa, BCI Competition III.

Studies	Methods	Year	k3b	k6b	l1b	Mean	std
Proposed Method	sr-mdrm		100	76.67	100	92.22	13.46
Zhang et al. [18]	Tsgsp	2018	99.2	67.2	96.5	87.63	17.74
Belwafi et al. [38]	wola-csp	2018	97.77	61.66	93.33	84.25	19.69
Conventional Method	csp		95.56	61.67	93.33	83.52	18.95
Horev et al. [25]	horev-mdrm	2017	95.56	68.33	85	82.96	13.72
Barachant et al. [23]	mdrm		96.66	60	88.33	81.66	19.21
Lotte and Guan [35]	srcsp	2011	96.67	53.33	93.33	81.11	24.11

**Table 6:** Classification accuracy (mean and standard deviation in percent) of the proposed approach and other approaches on Dataset IIa, BCIC IV.

Studies	Methods	Year	A01	A02	A03	A04	A05	A06	A07	A08	A09	Mean	Std
Proposed Method	Sr-mdrm		90.21	63.28	96.55	76.38	65.49	69.01	81.94	95.14	93.01	81.22	12.43
Gaur et al. [40]	ss-memdbf	2018	91.49	60.56	94.16	76.16	58.52	68.52	78.57	97.01	93.85	79.93	14.14
Barachant et al. [23]	Mdrm		91.61	57.03	90.21	73.61	73.94	68.31	75	95.14	90.21	79.45	12.92
Belwafi et al. [38]	Wola-csp	2018	86.81	63.19	94.44	68.75	56.25	69.44	78.47	97.91	93.75	78.85	15.15
Lotte and Guan. [35]	srcsp	2011	88.89	63.19	96.53	66.67	63.19	63.89	78.47	95.83	92.36	78.78	14.77
standard Method	csp		88.89	51.39	96.53	70.14	54.86	71.53	81.25	93.75	93.75	78.01	17.01
Raza et al. [41]	tlcsp${}_{1}$	2016	90.28	54.17	93.75	64.58	57.64	65.28	62.5	90.97	85.42	73.84	15.93
Raza et al. [41]	tlcsp${}_{2}$	2016	90.28	57.64	95.14	65.97	61.11	65.28	61.11	91.67	86.11	74.92	15.42
